# The Adequacy of Experimental Models and Understanding the Role of Non-coding RNA in Joint Homeostasis and Disease

**DOI:** 10.3389/fgene.2020.563637

**Published:** 2020-10-09

**Authors:** Letizia Penolazzi, Elisabetta Lambertini, Roberta Piva

**Affiliations:** ^1^Department of Biomedical & Specialty Surgical Sciences, University of Ferrara, Ferrara, Italy; ^2^University Center for Studies on Gender Medicine, University of Ferrara, Ferrara, Italy

**Keywords:** joint degeneration, molecular circuits, non-coding RNA, 3D *in vitro* models, drug discovery

## Introduction

RNA-mediated processes by non-coding RNA (ncRNAs) namely microRNAs, long ncRNAs and circular RNAs, as all epigenetic mechanisms are particularly sensitive to the effects of tissue microenvironment and environmental factors (Chisolm and Weinmann, 2018; Ning et al., 2019; Pagiatakis et al., 2019). In recent years research has focused on the development of smart cell culture *in vitro* systems one step closer to natural conditions, paying particular attention to the cellular microenvironment and cell culture conditions. 3D cell culture and co-culture systems based on cultivating a single cell population or different cell populations combined together, have found growing interest as useful tools to better understand cell biology and to offer more physiological relevant results by tightly controlling experimental parameters (Dhaliwal, 2015; Duval et al., 2017; Mirbagheri et al., 2019). The development of these *in vitro* models is a promising approach even if the limited availability of human tissue from which to obtain the cells have to take into account. Such an approach by using tissue specimens of human origin can allow the realization of suitable 3D *in vitro* models overcoming the limits of traditional 2D monolayer cell cultures, or expensive animal models that often cannot accurately recapitulate human etiopathogenesis and are not suited to develop novel drugs. It is widely recognized that in a 3D environment cells tend to be more subjected to morphological and functional changes differently to those grown in simplistic cellular monolayer. Another important issue is the methods matter regarding the employment of cell culture conditions that have to take into account the physiological parameters such as oxygen concentration, chemical and biophysical components.

As a whole, new 3D technologies recapitulating the essential aspect of the dynamic *in vivo* environment can meet the need of achieving stringent information to design adequate prevention strategies and to develop more effective therapeutics, including those based on RNA. Epigenetic investigation in all areas cannot underestimate this aspect. It is in fact important to take into consideration that ncRNAs display more tissue-specific expression patterns than protein-coding genes, and cell differentiation is particularly affected by fine tuning of ncRNAs level (Fatica and Bozzoni, 2014; Jiang et al., 2016; Ramón Y Cajal et al., 2019). Therefore, it follows that changes in the ncRNAs expression and functions, as well as their cellular delivery through extracellular vesicles (Di Liegro et al., 2017) may be particularly influenced by experimental conditions and methodological approach of carrying out the experiments.

This general premise is particularly appropriate for research aimed at studying joint homeostasis and skeletal pathologies, an area in which a great effort is being made to compare the effectiveness of different experimental models.

Bone and cartilage cells together with all other cells localized in the different types of skeletal joints are extremely sensitive to the effects of interstitial pH, oxygen levels, glucose concentration, mechanical stimuli, calcium content, and paracrine signaling.

Many skeletal degenerating conditions may be seen as the failure of soluble factors and epigenetic regulation to establish the correct microenvironment in bone and cartilage (van Meurs et al., 2019). Emerging evidence showed that ncRNAs, in particular miRNAs, are deregulated in many bone and joint diseases (Zhou et al., 2017; Wang et al., 2019; Wu et al., 2019; Zhu et al., 2019; Li et al., 2020; Song et al., 2020). A better understanding of RNA-mediated processes in epigenetics will help to deepen how bone and cartilage are damaged, providing novel repair strategies to restore joint function, as well as new insight on the pathogenesis of acquired and inherited bone or joint diseases (Houard et al., 2013; Sun et al., 2019; van Meurs et al., 2019).

However, artifacts arising from not satisfactory experimental models and/or contaminants in culture medium can produce incorrect information, weakening the biomedical relevance (Tosar et al., 2017).

Here we would like to express our opinion on (i) the importance of the adequacy of experimental models to understand the specific role of ncRNA, in joint/skeletal homeostasis, and (ii) the careful surveillance for warning signs of contamination and possible artifacts.

The final aim is to keep high attention and to stimulate discussion on the need to adopt effective tools to design appropriate human-oriented joint disease research focused on understanding the mechanisms of regulation of gene expression to develop specific RNA-based therapeutics.

## The Joint of Skeletal System: the Challenge of Developing Appropriate Experimental Models

Re-creating *in vitro* skeletal tissue microenvironment is a challenge that is engaging many researchers who are looking for the molecular mechanisms underlying tissue homeostasis maintenance for preclinical drug testing purposes. This challenge is especially hard for what concerns the joint of the skeletal system, due to its intrinsic biology. Currently, various strategies recapitulating the complex and dynamic three-dimensional (3D) environment experienced by cells *in vivo* are proposed by several research groups (Baker and Chen, 2012; Cassotta et al., 2020). These include the development of:

- various 3D—engineered systems based on co-cultures (Owen and Reilly, 2018),
- biomimetic materials resembling the natural extracellular environment combined with cells at different stage of maturation (Park et al., 2018),
- drug-releasing scaffolds (Zeng et al., 2019),
- bioreactors for dynamic culture conditions (Bicho et al., 2018),
- organ-on-a chip and integrated microfluidic culture platforms (Dai et al., 2019).

Methods and models listed above can offer great benefits especially when combined with a careful control of chemical and physical parameters (Bader et al., 2011; Baker, 2016). This means exposing the cellular system to:

- physiological oxygen concentration, by using specific cell culture solutions to create hypoxic environment. Primary cultures of chondrocytes or osteoblasts are typically carried out at normoxia (21% oxygen), while these cells are *in vivo* exposed to an oxygen gradient of 1–8%;
- mechanical stimuli, maintaining, for example, the cartilage integrity through the process of mechanotransduction obtained with suitable devices;
- refined and more physiologically relevant culture media, including serum.

The optimization and a wider use of these methods is essential. However, most of the data in the literature are still based on the use of cellular monolayers and conventional culture conditions. Yet it is well-known that, for example, isolated chondrocytes may change their phenotype after expansion in 2D monolayer cell culture (Caron et al., 2012).

Here we would like to draw attention to the need to create guidelines, in the near future, for the development of experimental models suitable for producing stringent data on joint homeostasis and new therapeutics development for joint degeneration. In particular, the common effort should be directed to identify experimental strategies and standardize reagents and protocols to optimize the results.

These reflections have the aim of ruling out misleading approaches, allowing the attribution of specific roles to molecular regulators, such as those supporting the RNA-mediated processes (Raman et al., 2018; Razmara et al., 2019; Zhang et al., 2019).

## Joint Degeneration and ncRNAs

Joint degeneration includes over 100 different types of arthritis conditions affecting usually the hips, knees, spine column, hands and feet (Xu and Li, 2020). The most widespread degenerative joint disease is the osteoarthritis (OA) which is one of the leading causes of disability in the middle-aged and elderly (Glyn-Jones et al., 2015). To date there is no cure for these disorders. The prevalence of this pathology is expected to increase in the coming years because the risk factors that favor it are inherent in today's society (aging, overweight and obesity, sedentary lifestyle, or uncontrolled sports practice) (Hoy et al., 2014; O'Neill et al., 2018; Zhang et al., 2019).

Joint degeneration process involves cartilage, bone and synovium (Xu and Li, 2020). It is only partially understood, and many aspects of the complex signaling between the joint forming cells and mechanisms driving cell fate decision inside the degenerated microenvironment have yet to be fully elucidated. In order to develop new therapeutics fighting joint degeneration, most studies are focusing on intra-articular injection of nucleic acid-based drugs including ncRNAs (Kawanishi et al., 2014; Wang et al., 2017; Cheng et al., 2018; Rai and Pham, 2018). Also in this case, improved *in vitro* experimental models are required to aid the identification of key regenerative ncRNAs that, once injected in a damaged joint, could potentially enhance endogenous repair and slow down the progression of joint tissues degeneration in all types of arthritis including after traumatic injury.

Abundant examples concerning the description of the expression and role of specific ncRNAs in the homeostasis of bone and cartilage tissues are present in the literature. It is difficult to choose the most significant and for this reason, please refer to recent reviews that gather the most important evidences (Endisha et al., 2018; Razmara et al., 2019; Li et al., 2020). Many of the papers cited in these reviews investigated the mechanism by which a ncRNA acts, by evaluating the variation in the expression of specific differentiation markers and the functionality of the cells following the silencing or overexpression of the ncRNA. In most cases, such an approach leads to conclusions about regulatory molecular circuits supported by specific epigenetic RNA-mediated processes. Most of the conclusions are certainly valid for the model used, but need to be confirmed in more sophisticated systems respecting biological complexity, and in view of a pre-clinical utility. In many cases a limited reproducibility was observed in studies aimed at elucidating the association of epigenetic signals with bone or joint phenotypes.

## The Risks That Can Be Taken Using Inappropriate Experimental Models

There are highly variable or unreliable conclusions about the role of ncRNAs in the maintenance of joint homeostasis or in contributing to the onset and progression of a joint disease. Literature often provides data that may have been influenced by the choice of partial and not fully representative experimental conditions considering the biochemical and structural complexity of the joint.

The main critical issues that we have identified are related to (1) the employment of unsuitable experimental models; (2) the occurrence of unperceived artifacts; (3) the subsequent impossibility to identify applications for personalized medicine.

Here are just a few examples. Recent studies focusing on lncRNA HOTAIR (HOX transcript antisense intergenic RNA) in cartilage and synovium suggest that it is involved in the regulation of the pathogenesis of OA and synovial inflammation, as HOTAIR silencing could restore collagen II and aggrecan expression (Chen et al., 2020). The analysis on extracellular matrix (ECM) degradation of human chondrocytes revealed that other lncRNAs such as H19, Nespas (a “sponge” targeting mir-291a-3p,−23a-3p,−24-3p,−196a-5p, and let-7a-5p), lncRNA-MSR (targeting miRNA-152) and lncRNA-CIR (targeting miR-27) display a similar role (Sun et al., 2019). In experiments like these, was the influence of physiological cues including mechanical loading or oxygen concentration considered to preserve the native epigenetic profile?

Deregulation of circSEMA4B, circRNA_104670, circ-4099, circ-GRB10, circVMA21 modulates the phenotype of nucleus pulposus cells inside the intervertebral disc (IVD), through sponging their target miRNAs and repressing (or derepressing) the corresponding downstream mRNAs (Li et al., 2020). With current evidence, is treatment of IVD degeneration through restoring the expression of downregulated circRNA or silencing of the aberrantly upregulated circRNAs feasible? To understand the difficulty of answering this question just think that the best way to achieve NP cell-specific delivery of ncRNA-based therapeutics remains undefined.

Regarding the risk of artifacts, it has been shown, for example, that the application of RNA-seq or “single-cell”—OMIC to profile ncRNAs from human cartilage biopsy and isolated chondrocytes suffer the influence of handling procedure and the choice of sample to include in the study (van Meurs et al., 2019). Careful handling and processing of cells is in fact critical to preserve the native epigenetic and expression profile. For instance, different results were obtained from live tissue donors or post-mortem, freshly isolated human chondrocytes or de-differentiated fibroblast like cells cultured in presence of chondrogenic inducers (Ajeekigbe et al., 2019).

We believe that efforts to develop and optimize particularly promising approaches such as those listed below should increase. For example, osteochondral plugs (from bovine, equine, and human specimens) which represent promising *ex-vivo* models for the study of joint diseases (Cope et al., 2019) are able to provide a reliable throughput model for proof of concept and mechanistic studies, applicable also to epigenetic analysis. Attractive alternative has been reached from multicompartmental modular bioreactors, microfluidic-based chip technology that developed “joint-on-a-chip,” and high-throughput single cell technologies to measure the epigenomic together genomic, transcriptomic or proteomic state of individul cells at high resolution (Groen et al., 2017; Hwang et al., 2017).

Most researchers rightly work with models that are easy to handle that allow to repeat the experiment numerous times to demonstrate the statistical significance of data: there is no doubt about the correctness of the method. However, in our opinion, it is important that studies relying on a large number of different tests have to be accompanied by “proof of concept” experiments by using methods we here mentioned to pave the way for the development of effective drugs.

In this regard, another aspect not to be overlooked is the added value that can derive from considering the data obtained from each single cell source and therefore from each individual donor/patient. Also with regard to epigenetics, research is heading toward an evolving concept based on a patient-oriented research. Research on RNA-mediated processes by specific ncRNAs in the joint homeostasis and the impact on potential development of new drugs requires also an analysis of the heterogeneity of different kinds of patients. It is important to mention that the best therapeutic decisions for the average patient are not necessarily the best decisions for an individual patient.

## Conclusions and Future Perspectives

The applicability of results from *in vitro* studies to *in vivo* situations, especially as regards the molecules involved in regulatory mechanisms, is directly dependent on the degree of similarity between the *in vitro* experimental condition and the *in vivo* environment. We believe a major effort and investment of time in this direction by the scientific community is necessary. The effort should be toward the improvement and the use of technology which allows cells from a specific donor to grow and behave *in vitro* in a manner that more closely represents that experienced by their native counterparts. This approach will likely have a significant impact on the understanding the real role of critical regulators of tissue homeostasis such as ncRNAs, and on improving drug discovery.

This objective can be achieved through different types of initiatives that connect the scientists who deal with joint homeostasis and disease, such as: (1) the creation of an international research Consortium dedicated to support the development and optimization of 3D cell culture models, (2) specific workshops for promoting the development of guidelines in order to minimize controversies on mechanisms of disease and potential therapeutic targets, (3) the creation of a blog managed by a joint scientific organization that promotes debate and where it is possible to meet the experts.

Certainly the biggest challenge is to convince those scientists to move from their already well-established 2D, and often successfully funded, cellular models. Therefore, we think that a critical point is represented by adequate funding policy that takes these issues into account and makes *ad hoc* funds available for studying and developing more relevant experimental models.

## Author Contributions

LP contributed the idea and the draft. EL contributed the overall structure and corrected the text. RP wrote the paper and conceived the study. All authors listed have made a substantial, direct and intellectual contribution to the work, and approved it for publication.

## Conflict of Interest

The authors declare that the research was conducted in the absence of any commercial or financial relationships that could be construed as a potential conflict of interest.
